# Fecal short-chain fatty acids and serum metabolites: the impact of COVID-19 infection on dialysis patients

**DOI:** 10.3389/fnut.2026.1772671

**Published:** 2026-02-19

**Authors:** Jiamin Duan, Jing Zhang, Changlin Li, Yuting Li, Duo Yu, Yuwei Chen, Qingli Yang, Xiaomeng Lin, Xudong Cai

**Affiliations:** 1 Nephrology Department, Ningbo Municipal Hospital of Traditional Chinese Medicine (TCM), Affiliated Hospital of Zhejiang Chinese Medical University, Ningbo, Zhejiang, China; 2 Nephrology Department, Ningbo Research Institute of Chinese Medicine, Ningbo Municipal Hospital of Traditional Chinese Medicine (TCM), Affiliated Hospital of Zhejiang Chinese Medical University, Ningbo, Zhejiang, China

**Keywords:** COVID-19, dialysis, long COVID, metabolomics, short-chain fatty acids

## Abstract

**Introduction:**

Patients undergoing dialysis are particularly susceptible to severe COVID-19 outcomes owing to pre-existing metabolic and immunological dysregulation, which may exacerbate clinical severity and elevate the risk of long COVID (LC). Nevertheless, the precise metabolic pathways implicated remain poorly characterized. This study aimed to characterize fecal short-chain fatty acids (SCFAs) and serum metabolomic signatures in dialysis patients with acute COVID-19 and to explore their association with LC.

**Methods:**

Targeted liquid chromatography–tandem mass spectrometry (LC–MS/MS) quantified fecal SCFAs in 27 infected patients and 28 non-infected controls, and untargeted gas chromatography–mass spectrometry (GC–MS)-based metabolomics profiled serum samples from 23 infected patients and all 40 controls in partially overlapping patient subsets, with repeat serum sampling at 3 months and stratification into LC and non-LC groups. Multivariate analyses, Kyoto Encyclopedia of Genes and Genomes (KEGG) pathway enrichment, and Pearson correlation analyses between differential metabolites and routine clinical indicators were performed.

**Results:**

Infected patients exhibited significantly lower fecal levels of six SCFAs, including propionate and butyrate, compared with controls. Serum metabolomics identified 54 infection-related differential metabolites enriched in amino acid, energy, carbohydrate, and nucleotide metabolism, and 77 LC-associated metabolites predominantly mapping to amino acid and energy pathways. Pearson correlation analysis showed that amino acids and energy-supporting metabolites (e.g., glutamine, aspartate, methionine, cystine, taurine) were inversely correlated with C-reactive protein, leukocyte and neutrophil counts, and aspartate aminotransferase, but positively correlated with albumin, serum potassium, and lymphocyte or eosinophil counts, whereas purine degradation products and organic acids (e.g., uric acid, hypoxanthine, pyruvate, glycolate) exhibited the opposite pattern.

**Discussion:**

COVID-19 infection in dialysis patients is associated with marked depletion of fecal SCFAs and broad perturbations of systemic metabolism, with persistent amino-acid-centered alterations among patients who develop LC. These findings offer a novel metabolic framework supporting the implementation of prolonged follow-up strategies to monitor and ameliorate persistent sequelae in this high-risk population.

## 1 Introduction

The coronavirus disease 2019 (COVID-19) pandemic, caused by the severe acute respiratory syndrome coronavirus 2 (SARS-CoV-2), has posed an unprecedented global health crisis, resulting in over 776.8 million confirmed COVID-19 cases and over 7 million confirmed deaths worldwide (1, 2). While the acute phase of the infection has been extensively characterized, a significant portion of convalescent individuals develop a debilitating post-viral syndrome known as Long COVID (LC), which affects an estimated 65 million people globally and manifests as heterogeneous symptoms including persistent fatigue, cognitive impairment, and respiratory issues (3, 4). Among this population, patients with end-stage renal disease (ESRD) on maintenance dialysis represent a particularly vulnerable subgroup because pre-existing immune dysfunction, chronic inflammation and profound metabolic disturbances jointly predispose them to severe acute COVID-19 and a higher burden of LC (5, 6). Elucidating the mechanisms that link SARS-CoV-2 infection to disease severity and long-term sequelae in this high-risk population is therefore a critical public health and clinical priority.

The clinical trajectory of COVID-19 is increasingly understood to be heavily influenced by the host's metabolic state, with perturbations in amino acid, energy, and lipid metabolism closely linked to disease severity and outcomes (7, 8). Metabolomics has emerged as a pivotal tool for uncovering these pathological shifts and identifying potential biomarkers (9). A particularly promising yet underexplored metabolic interface in COVID-19 is the gut-lung axis, with the gut microbiota and its functional output playing a crucial immunomodulatory role (10). Notably, short-chain fatty acids (SCFAs) depletion has been documented in both chronic kidney disease (CKD) populations and in individuals following COVID-19, suggesting a potential link to immune dysregulation and adverse clinical courses (11, 12). Straight-chain SCFAs primarily arise from the microbial fermentation of dietary carbohydrates, whereas branched-chain short-chain fatty acids (BCFAs) are mainly derived from the fermentation of branched-chain amino acids (BCAAs) (13). Despite these advances, critical knowledge gaps persist specifically concerning the dialysis population. The pre-existing uraemic milieu, characterized by gut dysbiosis, accumulation of uremic toxins and dialysis-related metabolic stress, likely creates a distinct metabolic landscape that interacts with SARS-CoV-2 differently from the general population (14, 15). However, the specific changes in gut microbiota–derived metabolites such as SCFAs and the accompanying systemic metabolic shifts after COVID-19 in dialysis patients remain largely undefined. Against this background, the present study sought to characterize fecal SCFAs profiles and serum metabolomic signatures in dialysis patients with and without acute COVID-19, and to explore their evolution in relation to LC, in order to provide a metabolic framework for risk stratification and targeted follow-up in this high-risk population.

## 2 Materials and methods

### 2.1 Study design and participants

**Figure 1:**
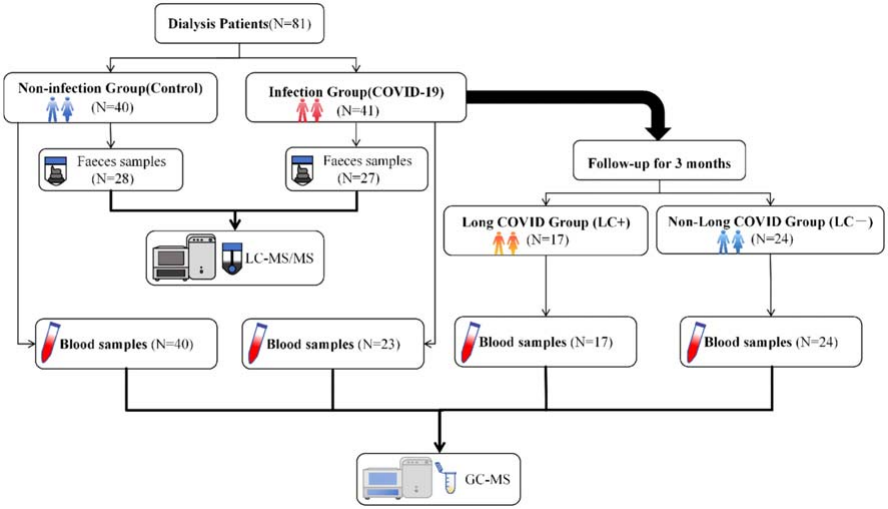
Flowchart of study enrollment, grouping, and sample collection.

This single-center observational study was conducted in the Department of Nephrology, Ningbo Hospital of Traditional Chinese Medicine. A total of 81 maintenance dialysis patients were enrolled between May 17 and August 31, 2023, including 41 hospitalized patients with COVID-19 (Infection Group) and 40 non-infected outpatient dialysis patients (Non-infection Group). Biological specimens for metabolomic profiling were collected after enrollment. Because of operational and clinical constraints, particularly the complex management of patients undergoing dialysis while acutely infected with COVID-19, sample availability differed between groups. Consequently, the final analytical dataset comprised fecal samples from 27 participants in the Infection Group and 28 participants in the Non-infection Group, serum samples from all 40 participants in the Non-infection Group, and serum samples from 23 participants in the Infection Group. All 41 patients in the Infection Group were followed for 3 months after hospital discharge. During the follow-up visits, clinical symptoms were recorded, and convalescent serum samples were collected (Figure 1). The study was approved by the Ethics Committee of Ningbo Hospital of Traditional Chinese Medicine and was registered at the Chinese Clinical Trial Registry (ChiCTR2300071494). Written informed consent was obtained from all participants or their legal representatives prior to inclusion. All data were anonymized to protect patient privacy, and the study was conducted in accordance with the ethical principles of the Declaration of Helsinki.

COVID-19 infection was diagnosed in accordance with the Diagnosis and Treatment Protocol for Novel Coronavirus Infection (Trial Version 10) issued by the National Health Commission of China, by meeting at least one etiological criterion: a positive result for SARS-CoV-2 nucleic acid by real-time reverse transcription–polymerase chain reaction on a respiratory (or other recommended) specimen, or a positive SARS-CoV-2 antigen test (or other virological evidence as specified in the 10th edition) indicating active infection (16). Dialysis patients were required to have CKD diagnosed according to Kidney Disease: Improving Global Outcomes guidelines and to be undergoing maintenance hemodialysis or peritoneal dialysis for ≥3 months (17, 18). LC was defined as the presence of one or more investigator-defined symptoms from an 11-item symptom list used in this study, persisting for ≥3 months after the acute phase of COVID-19, occurring in individuals with a history of probable or confirmed SARS-CoV-2 infection, and not better explained by an alternative diagnosis (19, 20). Specifically, we used a pre-specified 11-item symptom list based on contemporary LC case definitions and adapted to the dialysis population, corresponding exactly to the variables used in our LC symptom analysis dataset: (1) fatigue/malaise/weakness, (2) muscle/joint pain, (3) dyspnea, (4) cough, (5) chest pain, (6) palpitations, (7) sleep disturbance, (8) loss of taste/smell, (9) headache, (10) cognitive impairment (brain fog), and (11) memory problem. For each participant, the presence, duration and evolution of these symptoms were captured using a standardized questionnaire administered at the follow-up visit (≥3 months after acute infection). To minimize misclassification in this multimorbid dialysis population, all potential LC cases were independently adjudicated by two nephrologists who reviewed the medical records, dialysis charts and relevant laboratory and imaging data to assess whether the reported symptoms could be fully explained by pre-existing comorbidities (e.g., congestive heart failure, chronic obstructive pulmonary disease, anemia, depression) or by intercurrent acute illnesses (e.g., bacterial pneumonia, urinary tract infection). Participants whose symptoms were deemed fully attributable to an alternative diagnosis were not classified as LC. Any disagreements between the two reviewers were resolved by discussion and, when necessary, by consulting a third senior physician. The detailed 11-item symptom list is provided in Table 1.

**Table 1:** Eleven-item Long COVID symptom list, operational definitions and mapping to dataset variables in maintenance dialysis patients.

**Symptom No**.	**Symptom (questionnaire item)**	**Operational definition**
1	Fatigue/Malaise/ Weakness	Persistent tiredness, lack of energy, or generalized weakness not relieved by rest
2	Muscle/joint pain	Generalized myalgia or arthralgia lasting for ≥3 months after acute COVID-19
3	Dyspnea	Shortness of breath at rest or on exertion, new or clearly worse than pre–COVID-19 status
4	Cough	Persistent dry or productive cough persisting for ≥3 months after acute infection
5	Chest pain	Chest discomfort, tightness or pain not fully explained by known cardiac or pulmonary disease
6	Palpitations	Subjective awareness of rapid, strong or irregular heartbeat
7	Sleep disturbance	Difficulty falling asleep, staying asleep, or non-restorative sleep
8	Loss Of taste/smell	Anosmia, hyposmia, ageusia or dysgeusia that newly appeared or persisted after COVID-19
9	Headache	Recurrent or persistent headache that is new or clearly different from pre-existing patterns
10	Cognitive impairment (brain fog)	Difficulty concentrating, slowed thinking, problems with attention or mental clarity
11	Memory problem	Subjective short-term or long-term memory lapses reported by the patient or caregiver

All symptoms were assessed at the follow-up visit (≥3 months after acute COVID-19). For each symptom, responses were coded as 0 = absent and 1 = present during the preceding 3 months and representing a new symptom or a clear worsening compared with the pre–COVID-19 status.

Patients were excluded if they met any of the following criteria: (1) age <18 years; (2) pregnancy or lactation; (3) concurrent acute infections (e.g., bacterial pneumonia, urinary tract infection) or active autoimmune diseases (e.g., systemic lupus erythematosus, rheumatoid arthritis); (4) significant comorbidities including malignancy, severe hepatic insufficiency, or end-stage cardiopulmonary disease; (5) documented severe cognitive impairment or major psychiatric conditions; or (6) unavailability of complete clinical data or inability to comply with the follow-up protocol. To maintain diagnostic specificity for LC, wherein symptoms cannot be attributed to an alternative etiology, the following additional exclusion criteria were applied: (1) Onset of LC symptomatology occurring outside the predefined period from 2 weeks prior to 1 year following the initial COVID-19 diagnosis, ensuring a clear temporal relationship. (2) Symptoms that could be fully attributed to a documented pre-existing comorbidity (21).

### 2.2 Metabolomic sample preparation

Accurate metabolomic analysis relies heavily on proper sample collection and preparation. In this study, we followed rigorous protocols to minimize confounding variables and ensure high-quality data for both fecal SCFAs and serum metabolite analyses.

#### 2.2.1 Sample collection

Serum samples were collected from all participants after an 8-h fasting period, prior to the commencement of dialysis sessions. This protocol was chosen to reduce potential confounding effects from recent food intake or dialysis-related factors, both of which could significantly alter metabolic profiles. Blood was drawn using standard venipuncture procedures, and serum was isolated by centrifugation at 4°C. Samples were stored at −80°C until further processing (22). Fecal samples were obtained under comparable fasting conditions to serum samples, whenever feasible. Given that dietary intake and recent dialysis procedures can substantially influence gut-derived metabolites, fasting prior to sample collection helped minimize these potential confounders. Stool samples were immediately frozen at −80°C to preserve the integrity of the metabolites before analysis (23).

#### 2.2.2 Fecal SCFAs quantification by liquid chromatography–tandem mass spectrometry (LC–MS/MS)

SCFAs play a critical role in gut health and immune regulation, and their composition can reflect alterations in gut microbiota, which may be influenced by COVID-19 infection, particularly in dialysis patients. To quantify SCFAs, we utilized a TSQ Vantage triple quadrupole mass spectrometer (Thermo Fisher Scientific, USA) coupled with an UltiMate 3000 ultra-high performance liquid chromatography (UHPLC) system. These instruments provide high sensitivity and accuracy for the detection and quantification of SCFAs in complex biological matrices. Briefly, fecal samples were homogenized and extracted in 70% acetonitrile, and the resulting extracts were derivatized using 3-nitrophenylhydrazine (3-NPH) and carbodiimide hydrochloride (EDC·HCl) to improve detection sensitivity and specificity. The derivatized samples were then analyzed by LC–MS/MS using electrospray ionization (ESI) in negative ion mode and multiple reaction monitoring (MRM) for quantification of specific SCFAs, such as acetic, propionic, butyric, and valeric acids, along with their respective internal standard, d3-hexanoic acid (24).

Chromatographic separation was performed using a BDS HYPERSIL-C18 column (100 mm × 2.1 mm, 2.4 μm) maintained at 50°C. The mobile phase consisted of 0.1% formic acid in water (A) and 0.1% formic acid in acetonitrile (B), and a gradient elution program was applied to achieve effective separation of the derivatized SCFAs. The flow rate was maintained at 0.3 ml/min, and the injection volume was 5 μl (25). For MS detection, MRM transitions were used to identify and quantify each SCFA derivative; the spray voltage was set to 3200 V and the vaporizer temperature was maintained at 300°C. Collision energies were optimized for individual analytes, and the corresponding product ions were monitored to ensure high sensitivity and specificity. Table 2 below summarizes the optimized parameters for each SCFAs (26).

**Table 2:** Optimized Mass Spectrometric Parameters for Each Short-Chain Fatty Acid (SCFAs).

**Analyte**	**Precursor Ion (m/z)**	**Product Ion (m/z)**	**Collision Energy (eV)**	**S-Lens**
Acetic acid	194.1	137.1	20	40
Propionic acid	208.1	165.1	20	50
Butyric acid	222.1	137.1	22	65
Isobutyric acid	222.1	137.1	24	75
2-Methylbutyric acid	236.1	137.1	24	75
Valeric acid	236.1	137.1	24	75
Isovaleric acid	236.1	137.1	24	75
d3-Hexanoic acid (ISTD)	253.1	137.1	25	75

#### 2.2.3 Serum metabolomic profiling by gas chromatography–mass spectrometry(GC–MS)

Serum metabolites are crucial indicators of systemic metabolic changes and can provide insights into the effects of COVID-19 on dialysis patients. Serum samples (50 μl) were mixed with methanol containing 1, 2 ^13^C-myristic acid as an internal standard and subjected to a series of preparation steps, including centrifugation and drying under nitrogen. The dried serum extract was derivatized using methoxyamine and BSTFA (containing 1% TMCS), which facilitated the analysis of a broad range of metabolites by GC-MS (27).

Separation of derivatized metabolites was achieved on a TG-5MS capillary column (0.25 mm × 30 m, 0.25 μm) with an initial temperature of 60°C, ramped to 320°C over 14 min. Helium was used as the carrier gas at a flow rate of 1.2 ml/min, and the injection volume was 1 μL with a 20:1 split ratio (28). GC-MS analysis was performed with electron impact ionization at 70 eV, and mass spectra were acquired in full scan mode from m/z 50 to 500. The ion source temperature was maintained at 280°C, and the transfer line was heated to 250°C. This method enables the detection and quantification of a wide variety of metabolites, providing a comprehensive profile of the serum metabolome in dialysis patients (29). To ensure the reproducibility and reliability of the results, quality control (QC) samples were prepared by pooling aliquots from each participant's sample. The QC pool was processed and analyzed alongside the individual samples to assess the consistency of the analysis and minimize potential batch effects (30).

### 2.3 Statistical analysis

Statistical analysis was performed using the Storm Statistics platform (Storm Statistics - Storm Statistics, medsta.cn). The normality of continuous variables was assessed using the Shapiro-Wilk test, and homogeneity of variance was tested using Levene's test. Normally distributed data were presented as mean ± standard deviation (Mean ± SD), and comparisons between groups were performed using the independent samples t-test. Non-normally distributed data were presented as median (lower quartile, upper quartile) [M (Q1, Q3)], and group comparisons were conducted using the Mann-Whitney U test, with the test statistic reported as “Z”. Categorical data were presented as frequency (percentage) [*n* (%)], and inter-group differences were analyzed using the chi-square test (χ^2^ test). When the expected frequency was less than 5, Fisher's exact test was used. All statistical tests were two-tailed, with a significance level set at *P* < 0.05.

For metabolomics data analysis, MS-DIAL 4.9 software and the NIST library were used for compound identification. Raw chromatogram files generated by Xcalibur software were processed for peak extraction, deconvolution, compound identification, and peak alignment before being exported as.txt files containing metabolite information, including metabolite names, retention times, and m/z values (31). All data were uploaded to MetaboAnalyst 6.0, where median normalization was first applied, followed by log transformation for further standardization. The normalized and log-transformed data matrix was then exported and imported into SIMCA-P software, where principal component analysis (PCA) and orthogonal partial least squares discriminant analysis (OPLS-DA) were performed to visualize group separation and identify discriminative metabolic features. Differential metabolites were selected based on the variable importance in projection (VIP) score from OPLS-DA (VIP > 1.0) and *p*-values (*p* < 0.05). Kyoto Encyclopedia of Genes and Genomes (KEGG) pathway enrichment analysis was conducted using MetaboAnalyst 6.0. Pearson correlation analysis between clinical data and differential metabolites was performed using Origin 2024 (32).

## 3 Results

### 3.1 Clinical sample information

This study included a total of 81 dialysis patients, comprising 41 in the COVID-19 infection group and 40 in the non-infection control group. As summarized in Table 3, the two groups were well-matched in terms of age, gender distribution, and dialysis modality (all *P* > 0.05). However, the dialysis vintage was significantly longer in the infection group compared to the controls (*P* = 0.022). Patients in the infection group exhibited a more pronounced inflammatory response. This was characterized by significantly elevated levels of C-reactive protein (CRP), white blood cell count, and neutrophil count, alongside significantly decreased lymphocyte and eosinophil counts (all *P* < 0.05). In contrast, monocyte and basophil counts showed no significant differences between the groups. Serum albumin was significantly lower in the infection group (*P* < 0.001), while aspartate aminotransferase (AST) levels were significantly higher (*P* = 0.001). A trend toward lower hemoglobin was observed in the infection group, though it did not reach statistical significance (*P* = 0.077). No significant intergroup differences were found in alanine aminotransferase (ALT), serum creatinine, or blood urea nitrogen levels. Regarding electrolyte and dialysis efficacy, serum potassium levels were significantly lower in the infection group (*P* = 0.041). Furthermore, the Kt/V index, a marker of dialysis adequacy, was also significantly lower in patients with COVID-19 (*P* = 0.030).

**Table 3:** Comparison of baseline characteristics between the infection group and the non-infection group.

**Variables**	**Total (*n* = 81)**	**Non-infection group (*n* = 40)**	**Infection group (*n* = 41)**	**Statistic**	***P***
Age, M (Q1, Q3)	65.00 (54.00, 72.00)	65.00 (52.75, 71.25)	66.00 (55.00, 73.00)	*Z* = −0.66	0.511
**Gender**, ***n*** **(%)**				χ^2^=0.13	0.722
Male	47 (58.02)	24 (60.00)	23 (56.10)		
Female	34 (41.98)	16 (40.00)	18 (43.90)		
**Dialysis method**, ***n*** **(%)**				–	0.881
HD	49 (60.49)	23 (57.50)	26 (63.41)		
PD	26 (32.10)	14 (35.00)	12 (29.27)		
Mixed	6 (7.41)	3 (7.50)	3 (7.32)		
Dialysis duration (months), *M* (Q_1_, Q)	27.00 (15.00, 43.00)	21.00 (13.00, 35.25)	33.00 (18.00, 50.00)	*Z* = −2.29	0.022
CRP (mg/L), *M* (Q_1_, Q_3_)	6.90 (3.50, 23.30)	5.30 (3.03, 6.90)	23.30 (9.80, 43.50)	*Z* = −4.68	<001
WBC count (^*^10 /L), *M* (Q_1_, Q_3_)	5.80 (4.40, 7.10)	5.55 (4.20, 6.60)	6.50 (4.50, 8.00)	*Z* = −2.22	0.026
Neutrophil count (^*^10^9^/L), *M* (Q_1_, Q_3_)	4.00 (3.10, 5.20)	3.60 (2.98, 4.65)	4.80 (3.50, 6.10)	*Z* = −2.60	0.009
Monocyte count (^*^10^9^/L), *M* (Q_1_, Q_3_)	0.40 (0.30, 0.60)	0.40 (0.30, 0.50)	0.40 (0.30, 0.60)	*Z* = −1.19	0.234
Lymphocyte count (^*^10^9^/L), *M* (Q_1_, Q_3_)	1.10 (0.70, 1.40)	1.25 (0.88, 1.50)	0.80 (0.60, 1.30)	*Z* = −2.33	0.020
Eosinophil count (^*^10^9^/L), *M* (Q_1_, Q_3_)	0.10 (0.03, 0.19)	0.14 (0.05, 0.24)	0.08 (0.01, 0.15)	*Z* = −2.35	0.019
Basophil count (^*^10^9^/L), *M* (Q_1_, Q_3_)	0.02 (0.01, 0.03)	0.02 (0.01, 0.03)	0.02 (0.01, 0.02)	*Z* = −1.03	0.304
Hemoglobin (g/L), mean ± SD	107.84 ± 16.89	111.20 ± 16.13	104.56 ± 17.16	*t* = −1.79	0.077
AST(g/L), *M* (Q_1_, Q_3_)	17.00 (13.00, 21.00)	15.00 (12.00, 18.00)	18.00 (15.00, 24.00)	*Z* = −3.22	0.001
ALT (U/L), *M* (Q_1_, Q_3_)	13.00 (9.00, 20.00)	12.50 (8.00, 17.25)	14.00 (11.00, 21.00)	*Z* = −1.83	0.067
Albumin (g/L), mean ± SD	32.41 ± 4.87	34.21 ± 4.36	30.65 ± 4.74	*t* = −3.51	<.001
Creatinine (μ mol/L), mean ± SD	920.23 ± 248.46	924.02 ± 263.30	916.54 ± 236.31	*t* = −0.13	0.893
Blood urea nitrogen (μ mol/L), *M* (Q_1_, Q_3_)	19.34 (16.54, 24.47)	19.34 (16.66, 23.64)	19.21 (16.39, 27.95)	*Z* = −0.85	0.395
Serum potassium (mmol/L), Mean ± SD	4.27 ± 0.97	4.49 ± 0.99	4.05 ± 0.92	*t* = −2.08	0.041
Kt/V, Mean ± SD	1.59 ± 0.45	1.70 ± 0.40	1.48 ± 0.47	t = −2.21	0.030

*t, t*-test; Z, Mann-Whitney test; χ^2^, Chi-square test; Fisher exact.

SD, standard deviation; M, median; Q_1_, 1st quartile; Q_3_, 3st quartile.

Importantly, within the Infection Group, baseline demographic and clinical characteristics were broadly comparable between the overall cohort and the subsets that contributed fecal or serum samples, with no statistically significant differences in key variables (all *P* > 0.05; Table 4). This suggests that the biospecimen-based subgroups used for microbiome and metabolomic analyses are reasonably representative of the underlying infected dialysis population, thereby reducing the risk of systematic selection bias.

**Table 4:** Baseline characteristics of the overall infection group and the fecal and serum subsets.

**Variables**	**All infection patients (*n* = 41)**	**Fecal subset (*n* = 27)**	**Serum subset (*n* = 23)**	**Statistic**	***P***
Age, *M* (Q_1_, Q_3_)	66.00 (55.00, 73.00)	66.00 (55.00, 69.00)	66.00 (55.50, 73.00)	χ^2^ = 0.36^#^	0.837
**Gender**, ***n*** **(%)**				χ^2^ = 0.07	0.965
Male	23 (56.10)	16 (59.26)	13 (56.52)		
Female	18 (43.90)	11 (40.74)	10 (43.48)		
**Dialysis method**, ***n*** **(%)**					0.994
HD	26 (63.41)	18 (66.67)	16 (69.57)		
PD	12 (29.27)	7 (25.93)	6 (26.09)		
Mixed	3 (7.32)	2 (7.41)	1 (4.35)		
Dialysis duration (Months), *M* (Q_1_, Q_3_)	33.00 (18.00, 50.00)	33.00 (16.00, 54.00)	27.00 (16.00, 52.00)	χ^2^ = 0.11^#^	0.947
Hospitalization duration, *M* (Q_1_, Q_3_)	7.00 (6.00, 10.00)	7.00 (6.00, 10.00)	7.00 (5.50, 7.50)	χ^2^ = 2.60^#^	0.273
CRP (mg/L), *M* (Q_1_, Q_3_)	23.30 (9.80, 43.50)	16.60 (10.30, 42.30)	13.50 (3.30, 29.80)	χ^2^ = 1.77^#^	0.413
WBC count (^*^10^9^/L), *M* (Q_1_, Q_3_)	6.50 (4.50, 8.00)	6.60 (4.90, 9.00)	6.50 (4.45, 7.30)	χ^2^ = 0.93^#^	0.628
Neutrophil count (^*^10^9^/L), *M* (Q_1_, Q_3_)	4.80 (3.50, 6.10)	4.90 (3.55, 6.90)	4.80 (3.15, 5.30)	χ^2^ = 0.59^#^	0.743
Monocyte count (^*^10^9^/L), *M* (Q_1_, Q_3_)	0.40 (0.30, 0.60)	0.40 (0.30, 0.55)	0.40 (0.35, 0.55)	χ^2^ = 0.17^#^	0.919
Lymphocyte count (^*^10^9^/L), *M* (Q_1_, Q_3_)	0.80 (0.60, 1.30)	0.80 (0.60, 1.40)	0.80 (0.65, 1.15)	χ^2^ = 0.02^#^	0.99
Eosinophil count (^*^10^9^/L), *M* (Q_1_, Q_3_)	0.08 (0.01, 0.15)	0.08 (0.01, 0.17)	0.08 (0.01, 0.15)	χ^2^ = 0.38^#^	0.828
Basophil count (^*^10^9^/L), *M* (Q_1_, Q_3_)	0.02 (0.01, 0.02)	0.02 (0.01, 0.02)	0.02 (0.01, 0.02)	χ^2^ = 0.17^#^	0.919
Hemoglobin (g/L), Mean ± SD	104.56 ± 17.16	107.70 ± 17.47	105.13 ± 17.91	*F* = 0.28	0.759
AST(g/L), M (Q_1_, Q_3_)	18.00 (15.00, 24.00)	21.00 (15.00, 29.50)	21.00 (14.50, 27.00)	χ^2^ = 0.53^#^	0.765
ALT (U/L), M (Q1, Q3)	14.00 (11.00, 21.00)	17.00 (11.50, 24.00)	13.00 (10.50, 19.50)	χ^2^ = 1.38^#^	0.502
Albumin (g/L), mean ± SD	30.65 ± 4.74	31.09 ± 4.64	30.09 ± 4.22	*F* = 0.30	0.742
Creatinine (μ mol/L), M (Q_1_, Q_3_)	865.00 (753.00, 1023.00)	863.00 (748.00, 1059.00)	863.00 (762.00, 965.00)	χ^2^ = 0.05^#^	0.975
Blood urea nitrogen(μmol/L), *M* (Q_1_, Q_3_)	19.21 (16.39, 27.95)	19.21 (15.91, 28.05)	19.36 (16.24, 26.77)	χ^2^ = 0.04^#^	0.982
Serum potassium (mmol/L), Mean ± SD	4.05 ± 0.92	4.21 ± 0.89	4.19 ± 0.99	*F* = 0.28	0.755
Kt/V, mean ± SD	1.48 ± 0.47	1.45 ± 0.52	1.62 ± 0.47	*F* = 0.82	0.444

F, ANOVA; #, Kruskal-waills test, χ^2^, Chi-square test, Fisher exact.

SD, standard deviation, M, median, Q_1_, 1st quartile, Q_3_, 3st quartile.

### 3.2 Analysis of post-discharge symptoms in the infection group

All 41 patients in the Infection Group completed a 3-month outpatient follow-up after discharge. Based on the presence of persistent symptoms, they were categorized into a LC group (*n* = 17) and a Non-LC group (*n* = 24). As shown in Table 5, the two groups were comparable in terms of age, gender, dialysis vintage, and dialysis modality (all *P* > 0.05). However, significant differences were observed in clinical outcomes and dialysis efficacy. Furthermore, the Kt/V index, a marker of dialysis adequacy, was significantly lower in the LC group (*P* = 0.042). Symptom prevalence analysis revealed that fatigue (*P* < 0.001) and memory problems (*P* = 0.007) were reported significantly more frequently in the LC group. In contrast, the prevalence of other symptoms—including myalgia, dyspnea, cough, chest pain, palpitations, sleep disturbances, loss of taste/smell, headache, and cognitive impairment—did not differ significantly between the two groups (all *P* > 0.05).

**Table 5:** Comparison of clinical symptoms between the long COVID (LC) group and the non-LC group.

**Variables**	**Total (*n* = 41)**	**LC+(*n* = 17)**	**LC- (*n* = 24)**	**Statistic**	***P***
Age, *M* (Q_1_, Q_3_)	66.00 (55.00, 73.00)	67.00 (55.00, 73.00)	64.50 (55.75, 70.75)	*Z* = −0.44	0.662
**Gender, n (%)**				χ^2^=0.12	0.732
Male	23 (56.10)	9 (52.94)	14 (58.33)		
Female	18 (43.90)	8 (47.06)	10 (41.67)		
**Dialysis Method**, ***n*** **(%)**				–	0.687
HD	26 (63.41)	10 (58.82)	16 (66.67)		
PD	12 (29.27)	5 (29.41)	7 (29.17)		
Mixed	3 (7.32)	2 (11.76)	1 (4.17)		
Dialysis Duration (Months), m (Q_1_, Q_3_)	36.00 (21.00, 53.00)	40.00 (28.00, 55.00)	32.50 (19.50, 48.00)	*Z* = −1.06	0.290
Kt/V, Mean ± SD	1.48 ± 0.47	1.31 ± 0.35	1.61 ± 0.51	*t* = −2.10	0.042
Fatigue/malaise/weakness, *n* (%)	9 (21.95)	9 (52.94)	0 (0.00)	χ^2^ = 13.34	<001
Muscle/joint pain, *n* (%)	2 (4.88)	2 (11.76)	0 (0.00)	–	0.166
Dyspnea, n (%)	1 (2.44)	1 (5.88)	0 (0.00)	–	0.415
Cough, *n* (%)	3 (7.32)	3 (17.65)	0 (0.00)	χ^2^ = 2.34	0.126
Chest pain, *n* (%)	1 (2.44)	1 (5.88)	0 (0.00)	–	0.415
Palpitations, *n* (%)	1 (2.44)	1 (5.88)	0 (0.00)	–	0.415
Sleep disturbance, *n* (%)	3 (7.32)	3 (17.65)	0 (0.00)	χ^2^ = 2.34	0.126
Loss Of taste/smell, *n* (%)	2 (4.88)	2 (11.76)	0 (0.00)	–	0.166
Headache, n(%)	2 (4.88)	2 (11.76)	0 (0.00)	–	0.166
Cognitive impairment (Brain fog), *n* (%)	2 (4.88)	2 (11.76)	0 (0.00)	–	0.166
Memory problem, *n* (%)	6 (14.63)	6 (35.29)	0 (0.00)	χ^2^ = 7.30	0.007

Z, Mann-Whitney test; χ^2^, Chi-square test, Fisher exact.

M, median; Q_1_, 1st Quartile; Q_3_, 3st Quartile.

### 3.3 Analysis of fecal short-chain fatty acids

**Figure 2:**
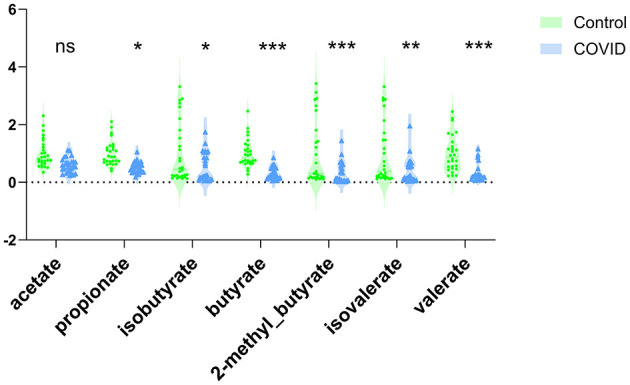
COVID-19 infection is associated with reduced fecal SCFAs levels in maintenance dialysis patients (Infection Group, *n* = 27; Non-infection Group, *n* = 28). *P < 0.05; **P < 0.01; ***P < 0.001; ns, not significant.

Analysis of fecal SCFAs concentrations revealed significant alterations in COVID-19 infected dialysis patients (Infection Group, *n* = 27) compared to their non-infected counterparts (Non-infection Group, *n* = 28) (Figure 2). Specifically, the infected group demonstrated markedly lower levels of multiple SCFAs, including propionic acid, isobutyric acid, butyric acid, 2-methylbutyric acid, isovaleric acid, and valeric acid (all *P* < 0.05). In contrast, the difference in acetic acid levels between the two groups did not reach statistical significance (*P* > 0.05).

### 3.4 Serum metabolomics analysis

#### 3.4.1 Discriminative analysis of serum metabolites in dialysis patients with COVID-19 infection

**Figure 3:**
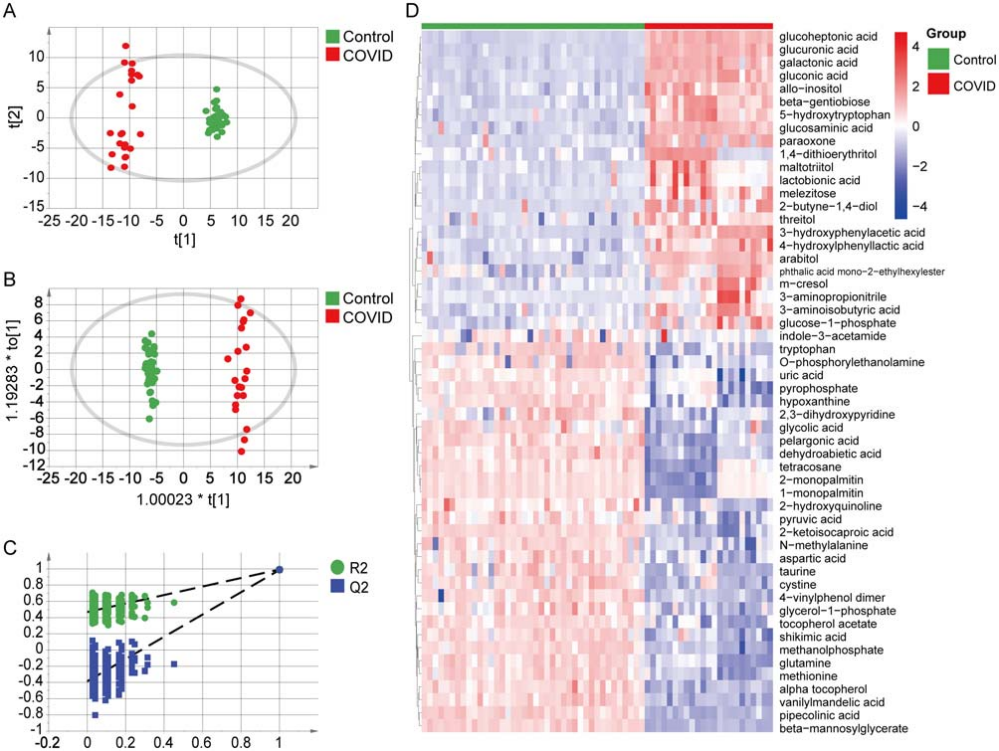
Baseline serum metabolomic profiling and discriminatory analysis between Infection and Non-infection groups (GC–MS). **(A)** Scatter plot of the infection group and non-infection group in the principal component analysis (PCA) model; **(B)** Scatter plot of the two groups in the orthogonal partial least-squares discriminant analysis (OPLS-DA) model, showing robust discrimination (*R*^2^*Y* = 0.996, *Q*^2^*Y* = 0.989); **(C)** Permutation test repeated 200 times, in which the original R^2^Y/Q^2^Y values exceed those of the permuted models and a Q2 intercept < 0.05 indicates no overfitting. **(D)** Heatmap of 54 metabolites differing between Infection and non-infection groups (VIP > 1.0, *P* < 0.05).

This study conducted a PCA analysis of the serum metabolites in the infected group and the non-infected group, as shown in Figure 3A. The results revealed that the samples within each group were well-clustered, while there was a clear separation between the groups. This indicates that the metabolic characteristics of samples within each group were relatively consistent, whereas the metabolic profiles between the groups differed significantly, suggesting that the infection status had a significant impact on the overall metabolic profile of the patients. To eliminate the influence of within-group differences and other unrelated noise factors, OPLS-DA was applied for statistical analysis of the patient samples (Figure 3B). The results showed that the infection and non-infection groups exhibited good fitting and predictive abilities in the established data model (*R*^2^*Y* = 0.996, *Q*^2^*Y* = 0.989). Further validation of the model was performed using 200 permutation tests. As shown in Figure 3C, the original R^2^Y and Q^2^Y values were higher than the random values of the permutation models, with the Q2 intercept less than 0.05, indicating that the model was not overfitted. By combining the OPLS-DA results with the Variable Importance in Projection (VIP) values of the corresponding metabolites, and setting VIP >1 and *P* < 0.05 as criteria, 54 differential metabolites were identified, as listed in Table 6, with their relative abundances visualized in a heatmap (Figure 3D).

**Table 6:** Differential metabolites between the infection and non-infection groups and their relative abundances.

**Compounds**	**Rt/min**	**m/z**	**VIP**	***P***	**Mean difference**
1,4-dithioerythritol	16.61	221.06	1.08	2.44E−07	−2.257
1-monopalmitin	13.40	239.19	1.34	7.28E−07	1.027
2,3-dihydroxypyridine	7.31	240.06	1.10	2.33E−02	−1.009
2-butyne-1,4-diol	6.39	147.41	1.10	9.12E−10	−2.714
2-hydroxyquinoline	8.14	202.01	1.13	4.69E−03	1.028
2-ketoisocaproic acid	6.10	200.08	1.42	9.97E−16	1.004
2-monopalmitin	13.25	218.10	1.32	2.02E−13	1.024
3-aminoisobutyric acid	7.86	174.10	1.20	6.89E−09	−1.884
3-aminopropionitrile	7.44	239.03	1.07	1.90E−02	−1.044
3-hydroxyphenylacetic acid	8.94	252.13	1.46	1.69E−03	−1.290
4-hydroxylphenyllactic acid	10.39	179.03	1.26	3.31E−18	−1.171
4-vinylphenol dimer	8.57	192.05	1.19	4.14E−02	1.008
5-hydroxytryptophan	10.14	290.04	1.37	4.43E−05	−1.185
Allo-inositol	11.12	318.08	1.40	7.86E−08	−1.983
Alpha tocopherol	15.81	237.11	1.45	6.16E−05	1.006
Arabitol	9.46	217.04	1.32	2.57E−08	−1.168
Aspartic acid	8.21	232.06	1.23	1.28E−02	−1.025
Beta-gentiobiose	14.69	361.06	1.42	1.81E−06	−3.235
Beta-mannosylglycerate	10.18	217.04	1.23	7.97E−04	−1.425
Cysteine	12.28	218.08	1.23	7.14E−05	1.018
Dehydroabietic acid	12.74	239.14	1.44	4.41E−02	−1.000
Galactonic acid	9.74	292.03	1.50	3.88E−16	−1.575
Glucoheptonic acid	9.05	217.04	1.56	2.63E−36	−1.837
Gluconic acid	11.00	333.04	1.51	4.11E−36	−1.832
Glucosaminic acid	8.40	147.03	1.41	1.22E−24	−1.192
Glucose-1-phosphate	9.81	217.07	1.09	2.08E−04	−1.197
Glucuronic acid	10.67	333.02	1.56	5.48E−22	−1.666
Glutamine	9.70	156.09	1.38	9.20E−13	1.021
Glycerol-1-phosphate	9.67	299.00	1.21	1.57E−05	−1.019
Glycolic acid	5.01	147.04	1.09	3.00E−09	−1.008
Hypoxanthine	9.94	265.07	1.24	1.28E−02	−1.017
Indole-3-acetamide	8.64	290.04	1.27	5.52E−04	−1.181
Lactobionic acid	13.90	204.06	1.14	1.56E−04	−1.281
Maltotriitol	14.03	203.98	1.23	2.49E−10	−1.363
m-cresol	5.65	165.02	1.08	1.50E−06	1.000
Melezitose	14.25	361.02	1.25	3.46E−08	−1.282
Methanolphosphate	5.86	241.00	1.45	1.11E−14	1.009
Methionine	8.23	176.06	1.43	2.78E−14	−1.030
N-methylalanine	5.79	130.07	1.10	2.09E−11	1.001
O-phosphorylethanolamine	9.82	299.03	1.03	3.58E−02	−1.004
Paraoxone	7.97	218.46	1.24	7.60E−15	−1.910
Pelargonic acid	7.12	215.14	1.41	2.47E−07	1.005
Phthalic acid mono-2-ethylhexylester	4.96	221.04	1.12	4.92E−03	−1.037
Pipecolinic acid	6.98	156.12	1.50	1.22E−05	1.025
Pyrophosphate	9.14	450.97	1.20	3.79E−07	1.013
Pyruvic acid	4.81	174.03	1.05	2.56E−05	−1.013
Shikimic acid	9.87	204.09	1.26	1.18E−05	1.016
Taurine	9.21	326.04	1.17	6.34E−03	1.017
Tetracosane	8.03	85.10	1.33	2.64E−03	1.030
Threitol	8.14	217.00	1.04	1.12E−06	−1.552
Tocopherol acetate	15.79	311.18	1.28	1.25E−14	1.015
Tryptophan	12.01	202.00	1.03	1.37E−04	1.017
Uric acid	11.41	441.03	1.09	4.10E−02	−1.001
Vanilylmandelic acid	19.05	297.16	1.46	6.66E−10	−1.003

Definition of mean difference: the last column reports the difference in mean relative abundance between groups (non-infection group minus infection group) calculated on the median-normalized and log-transformed data. Positive values indicate higher levels in the Non-infection group, whereas negative values indicate higher levels in the infection group.

**Figure 4:**
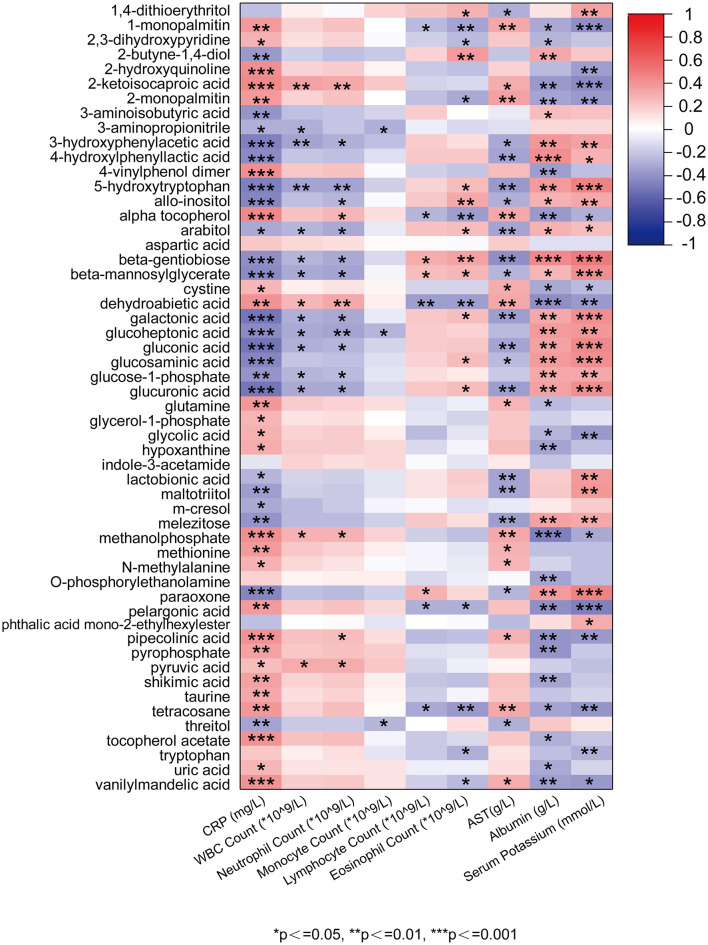
Pearson correlation heatmap between differential serum metabolites (y-axis) and clinical blood indices (x-axis). Colors indicate Pearson's correlation coefficients.

We used Pearson correlation analysis to examine the relationships between the 54 differential metabolites and routine clinical indicators (Figure 4). Most metabolites were significantly associated with at least one clinical parameter and clustered into two broadly opposing correlation patterns. Overall, amino acids and energy-supporting metabolites (such as glutamine, aspartic acid, methionine, cystine, taurine, and several sugar alcohols and carbohydrate intermediates) tended to show negative correlations with inflammatory markers, including CRP, leukocyte and neutrophil counts and AST, and positive correlations with albumin, serum potassium, and lymphocyte or eosinophil counts. These associations suggest that depletion of these substrates tracks with a more inflamed, catabolic and immunologically compromised state in infected dialysis patients. In contrast, purine degradation products and organic acids (including uric acid, hypoxanthine, pyruvic acid, glycolic acid and related metabolites) generally exhibited positive correlations with CRP, leukocyte and neutrophil counts and inverse correlations with albumin and serum potassium, indicating that their accumulation parallels neutrophil-driven inflammation, cellular injury and impaired nutritional status. Taken together, this bidirectional correlation pattern is compatible with a working model in which acute SARS-CoV-2 infection in dialysis patients is accompanied by intensive consumption of amino acids and related intermediates to support acute-phase protein synthesis and immune cell function, whereas concomitant cellular stress and a shift toward glycolytic energy metabolism are associated with enhanced purine catabolism and the build-up of organic acids. Although these findings are observational and do not establish causality, they provide a biologically plausible framework linking the identified metabolite clusters to systemic inflammation, protein–energy wasting and immune dysregulation in this high-risk population.

#### 3.4.2 Discriminative analysis of serum metabolites in dialysis patients affected by LC after discharge

**Figure 5:**
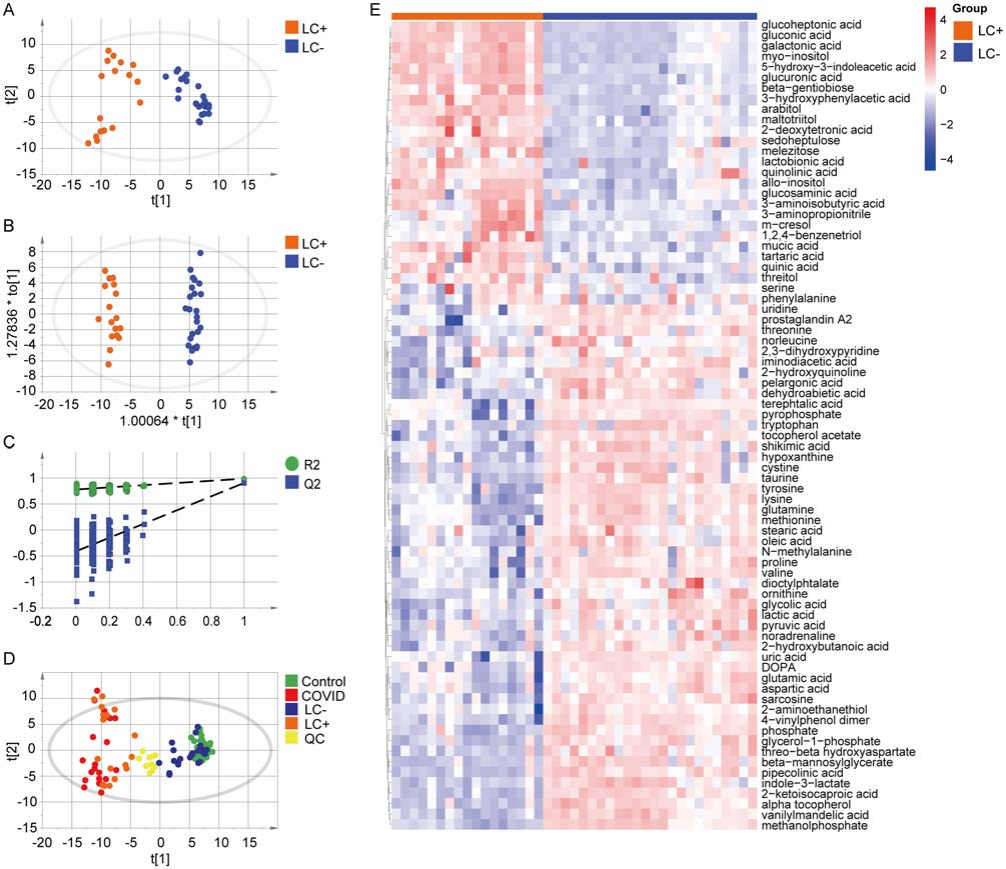
Serum metabolomic discrimination of Long COVID after discharge in dialysis patients. **(A)** PCA score plot comparing the Long COVID and non–Long COVID groups, showing within-group clustering and inter-group separation. **(B)** OPLS-DA score plot demonstrating robust discrimination with strong model fit and predictability (*R*^2^*Y* = 1.000, *Q*^2^*Y* = 0.896). **(C)** Permutation test (*n* = 200) confirming model validity without overfitting (Q2 intercept < 0.05). **(D)** Score plot including Control, COVID, LC–, LC+ and QC samples, with tightly clustered QCs and distinct group distributions. **(E)** Heatmap of 77 differential metabolites identified between groups (VIP > 1.0 and adjusted *P* < 0.05), with values median-normalized, log-transformed, and autoscaled prior to visualization.

This study performed a PCA analysis on the serum metabolites of patients in the LC group and the non-LC group, as shown in Figure 5A. The results indicated that the samples within each group were well-clustered, with a clear separation between the groups, suggesting that the presence or absence of LC significantly impacted the overall metabolic profile of the patients. To further assess data quality and the overall distribution of different cohorts, a score plot including Control, COVID, LC–, LC+ and QC samples was generated (Figure 5D). The QC samples clustered tightly, indicating good instrumental stability and high analytical reproducibility. Meanwhile, the different groups showed distinct spatial distributions: Control and COVID samples were largely separated, and LC+ samples exhibited an obvious shift away from LC–/Control, supporting that LC status after discharge was associated with a persistent metabolic deviation in dialysis patients. Statistical analysis of the two groups' samples was further conducted using OPLS-DA (Figure 5B), and the results demonstrated good fitting and predictive capabilities of the established data model for both groups (*R*^2^*Y*=1.000, *Q*^2^*Y*=0.896). To validate the model, 200 permutation tests were performed, as shown in Figure 5C. The original R^2^Y and Q^2^Y values were higher than the random values from the permutation models, with the Q2 intercept being less than 0.05, indicating no overfitting of the model. The OPLS-DA results were combined with the VIP values of the corresponding metabolites, and differential metabolites were selected based on the criteria of VIP > 1 and *P* < 0.05. In total, 77 differential metabolites were identified, as shown in Table 7, with their relative abundances visualized in a heatmap (Figure 5E).

**Table 7:** Differential metabolites between the long COVID and non-long COVID groups and their relative abundances.

**Compounds**	**Rt/min**	**m/z**	**VIP**	***P***	**Mean difference**
1,2,4-benzenetriol	8.74	342.09	1.16	4.12E−04	– 2.27
2,3-dihydroxypyridine	7.31	240.06	1.13	3.61E−03	−1.23
2-aminoethanethiol	4.02	174.08	1.10	1.96E−04	1.00
2-deoxytetronic acid	7.63	233.04	1.23	6.81E−06	−7.65
2-hydroxybutanoic acid	5.44	147.03	1.39	3.16E−08	4.23
2-hydroxyquinoline	8.14	202.01	1.22	1.22E−06	3.41
2-ketoisocaproic acid	6.10	200.08	1.65	1.11E−14	1.85
3-aminoisobutyric acid	7.86	174.10	1.24	8.35E−06	−16.99
3-aminopropionitrile	7.44	239.03	1.31	3.19E−06	−7.55
3-hydroxyphenylacetic acid	8.94	252.13	1.46	4.21E−10	−5.67
4-vinylphenol dimer	8.57	192.05	1.56	3.19E−11	1.45
5-hydroxy-3-indoleacetic acid	11.94	290.10	1.64	2.84E−14	−4.50
Allo-inositol	11.12	318.08	1.41	3.60E−08	−2.36
Alpha tocopherol	15.81	237.11	1.54	9.07E−11	1.36
Arabitol	9.46	217.04	1.55	1.02E−11	−4.25
Aspartic acid	8.21	232.06	1.25	1.17E−05	1.44
Beta-gentiobiose	14.69	361.06	1.56	6.61E−11	−5.25
Beta-mannosylglycerate	10.18	217.04	1.50	8.88E−11	2.91
Cystine	12.28	218.08	1.22	1.94E−05	4.13
Dehydroabietic acid	12.74	239.14	1.45	7.76E−08	−1.08
Dioctylphtalate	13.29	149.02	0.98	2.37E−04	1.87
DOPA	8.90	218.04	1.28	3.46E−06	1.68
Galactonic acid	9.74	292.03	1.67	1.06E−15	−4.60
Glucoheptonic acid	9.05	217.04	1.71	5.52E−18	−4.42
Gluconic acid	11.00	333.04	1.65	2.15E−14	−4.74
Glucosaminic acid	8.40	147.03	1.36	1.32E-07	−4.32
Glucuronic acid	10.67	333.02	1.69	1.32E−17	−5.74
Glutamic acid	8.81	246.05	1.18	2.90E−05	1.40
Glutamine	9.70	156.09	1.44	7.51E−08	4.12
Glycerol-1-phosphate	9.67	299.00	1.49	7.86E−10	1.37
Glycolic acid	5.01	147.04	1.37	1.68E−07	1.04
Hypoxanthine	9.94	265.07	1.29	1.29E−06	2.47
Iminodiacetic acid	7.94	232.06	1.18	9.57E−05	1.03
Indole-3-lactate	11.98	202.03	1.47	1.70E−09	1.78
Lactic acid	4.90	146.96	1.48	4.49E−09	1.04
Lactobionic acid	13.90	204.06	1.18	1.76E−05	−3.65
Lysine	10.50	174.05	1.52	1.37E−10	2.27
Maltotriitol	14.03	203.98	1.46	1.58E−10	−8.61
m-cresol	5.65	165.02	1.25	1.24E−05	−16.39
Melezitose	14.25	361.02	1.21	1.41E−05	−15.52
Methanolphosphate	5.86	241.00	1.69	3.34E−16	2.27
Methionine	8.23	176.06	1.45	1.26E−07	1.89
Mucic acid	10.19	292.05	1.47	4.73E−10	−4.24
Myo-inositol	11.43	304.99	1.64	9.02E−14	−3.22
N-methylalanine	5.79	130.07	1.02	8.32E−04	1.56
Noradrenaline	6.51	174.11	1.22	2.73E−06	1.04
Norleucine	5.88	158.09	1.12	4.61E−05	41.82
Oleic acid	11.84	95.14	1.32	1.12E−05	1.24
Ornithine	8.77	142.11	1.41	1.27E−08	3.25
Pelargonic acid	7.12	215.14	1.31	6.21E−07	−1.05
Phenylalanine	8.91	218.04	1.27	2.05E−05	1.43
Phosphate	6.57	298.95	1.45	3.07E−10	1.15
Pipecolinic acid	6.98	156.12	1.58	1.05E−13	3.98
Proline	6.74	142.04	1.15	6.13E−05	1.19
Prostaglandin A2	16.02	118.64	1.03	7.17E−05	1.33
Pyrophosphate	9.14	450.97	1.25	1.22E−05	3.06
Pyruvic acid	4.81	174.03	1.04	6.10E−04	1.08
Quinic acid	10.24	345.10	1.05	1.74E−04	−2.97
Quinolinic acid	9.50	147.07	1.03	5.81E−04	−1.96
Sarcosine	5.24	116.02	1.40	1.13E−08	1.33
Sedoheptulose	9.38	217.07	1.29	2.61E−06	−1.93
Serine	7.16	204.04	1.55	2.87E−11	1.60
Shikimic acid	9.87	204.09	1.22	1.78E−05	1.57
Stearic acid	11.85	116.89	1.03	5.86E−03	1.24
Tartaric acid	8.96	292.09	1.19	1.22E−05	−4.43
Taurine	9.21	326.04	1.43	8.17E−09	4.65
Terephtalic acid	13.55	311.17	1.08	2.40E−04	1.65
Threitol	8.14	217.00	1.08	2.07E−05	−13.33
Threo-beta hydroxyaspartate	7.91	218.03	1.40	1.43E−08	1.45
Threonine	7.35	218.06	1.11	2.42E−05	1.05
Tocopherol acetate	15.79	311.18	1.35	3.34E−08	2.68
Tryptophan	12.01	202.00	1.20	5.40E−06	4.32
Tyrosine	10.60	218.01	1.43	1.18E−08	1.79
Uric acid	11.41	441.03	1.19	4.05E−05	1.84
Uridine	12.97	217.09	1.06	4.54E−04	−1.11
Valine	6.13	144.05	1.34	1.17E−07	1.70
Vanilylmandelic acid	19.05	297.16	1.63	1.61E−13	1.69

Definition of mean difference: The last column reports the difference in mean relative abundance between groups (Non-LC group minus LC Group) calculated on the median-normalized and log-transformed data. Positive values indicate higher levels in the Non-LC Group, whereas negative values indicate higher levels in the LC Group.

#### 3.4.3 Metabolic pathway enrichment analysis

**Figure 6:**
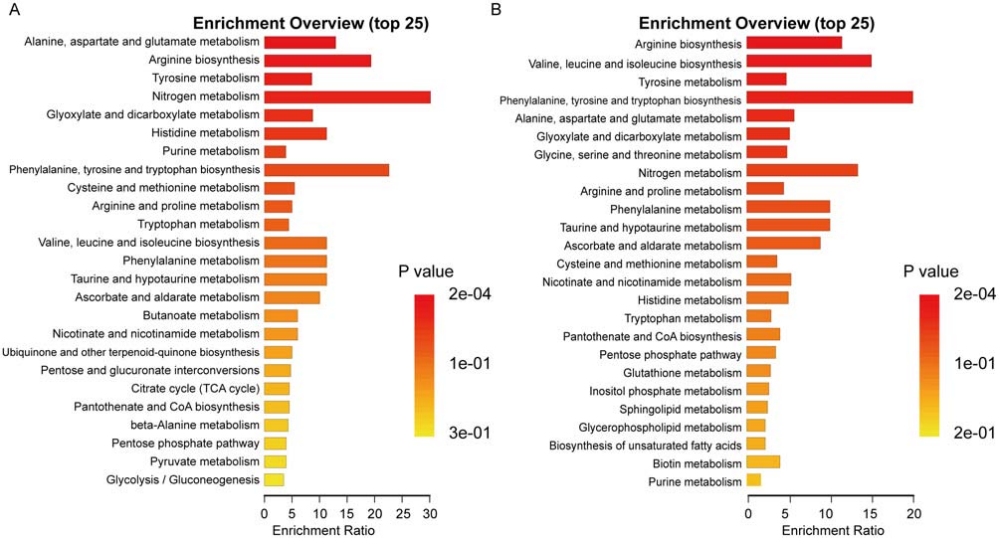
KEGG pathway enrichment of differential serum metabolites. **(A)** Top enriched metabolic pathways for the 54 differential metabolites associated with acute COVID-19 infection in dialysis patients. **(B)** Top enriched metabolic pathways for the 77 differential metabolites associated with Long COVID after discharge.

To explore the potential pathways affected by COVID-19 infection in dialysis patients, pathway enrichment analysis was performed on the 54 differential metabolites selected. The results showed that the top ten most significant pathways were primarily related to amino acid metabolism (Alanine, aspartate and glutamate metabolism, Arginine biosynthesis, Tyrosine metabolism, Histidine metabolism, Phenylalanine, tyrosine and tryptophan biosynthesis, Cysteine and methionine metabolism, Arginine and proline metabolism), energy metabolism (Nitrogen metabolism), carbohydrate metabolism (Glyoxylate and dicarboxylate metabolism), and nucleotide metabolism (Purine metabolism). The pathway enrichment analysis results are shown in Figure 6A. Subsequently, pathway enrichment analysis was conducted on the 77 differential metabolites associated with Long COVID, as shown in Figure 6B. The top ten significantly enriched pathways were mainly related to amino acid metabolism (Arginine biosynthesis, Valine, leucine and isoleucine biosynthesis, Tyrosine metabolism, Alanine, aspartate and glutamate metabolism, Phenylalanine, tyrosine and tryptophan biosynthesis, Glycine, serine and threonine metabolism, Arginine and proline metabolism, Phenylalanine metabolism), energy metabolism (Nitrogen metabolism), and carbohydrate metabolism (Glyoxylate and dicarboxylate metabolism). Importantly, given that humans lack the enzymatic machinery for the *de novo* synthesis of these metabolites, these serum enrichment signals likely reflect alterations in gut microbiota-driven production and the subsequent systemic absorption of these microbial products in the context of COVID-19-related dysbiosis.

## 4 Discussion

COVID-19 superimposed on end-stage kidney disease produced a characteristic pattern of gut and systemic metabolic disruption in this cohort of dialysis patients. Fecal profiles showed a marked loss of microbiota-derived SCFAs, while serum metabolomics revealed extensive reprogramming of amino acid, energy and carbohydrate metabolism during acute infection, and a persistent amino-acid–centered signature among patients who subsequently developed LC. Notably, pathway enrichment analysis suggested significant changes in pathways annotated as essential amino acid biosynthesis, including valine, leucine and isoleucine biosynthesis as well as phenylalanine, tyrosine and tryptophan biosynthesis. Because the end products of these pathways are essential amino acids that humans cannot synthesize *de novo*, enrichment signals assigned to these biosynthetic pathways in serum are unlikely to represent host-intrinsic biosynthetic activity. Instead, they more plausibly reflect altered metabolic functions of the gut microbiota and microbial contributions to the circulating amino acid pool. This interpretation is consistent with prior work showing that the gut microbiota can synthesize and metabolize essential amino acids, and that COVID-19 is associated with marked shifts in the gut microbiota and related metabolites, including branched-chain and aromatic amino acids (33, 34). This inference is further supported by the pronounced disruption of the fecal SCFA profile observed in our patients. Because SCFAs are key indicators of commensal microbial homeostasis, their depletion points to substantial perturbation of the gut ecosystem. Together, these observations suggest that SARS-CoV-2 infection may further remodel an already vulnerable gut ecosystem in dialysis patients, thereby contributing to systemic serum metabolomic signatures. Correlation analysis further linked these metabolic derangements to inflammatory burden, nutritional status and immune cell counts, highlighting tight coupling between the metabolic and clinical phenotype in this highly vulnerable population. Amino acids and energy-supporting intermediates, including glutamine, aspartate, methionine, cystine, and taurine, were inversely associated with CRP, leukocyte and neutrophil counts, and AST, and positively associated with albumin, potassium, and lymphocyte or eosinophil counts. In contrast, purine degradation products and selected organic acids, including uric acid, hypoxanthine, pyruvate, and glycolate, showed the opposite pattern. These associations are cross-sectional and do not establish causality or directionality. however, they align with immunometabolic frameworks in which sustained inflammatory signaling increases the demand for amino acids that support biosynthesis, redox balance, and energetic needs, while immune activation and cellular stress are frequently accompanied by altered glycolytic flux and increased purine turnover (35, 36). In the context of uremia-related metabolic inflexibility, such infection-associated reprogramming may have amplified clinical consequences.

Dialysis patients generally exhibit an inherent “immune paralysis” state, characterized by lymphocyte depletion and impaired T-cell function, leading to a weakened immune response to viral infections and increased susceptibility to pathogens such as SARS-CoV-2 (37, 38). Once an infection occurs, these patients often experience an excessive inflammatory response due to the imbalance in immune regulation, which is consistent with the clinical data from this study (blood routine and CRP) (39). Additionally, dialysis patients typically have gut dysbiosis and impaired intestinal barrier function, contributing to a mild but persistent chronic inflammatory state (40). The SARS-CoV-2 virus can invade the intestinal mucosa through its receptor, angiotensin-converting enzyme 2 (ACE2), further exacerbating gut microbiota dysbiosis and profoundly affecting the composition of gut microbiota metabolites (41, 42). SCFAs are important metabolites produced by gut microbiota fermentation of dietary fibers, playing a central role in regulating host immune responses and inflammatory reactions (43). Their anti-inflammatory mechanisms include: first, as histone deacetylase (HDAC) inhibitors, especially butyrate, which can promote the differentiation and function of regulatory T cells (Treg) with anti-inflammatory functions, thereby maintaining immune homeostasis (44). Second, by activating G protein-coupled receptors (GPCRs), SCFAs inhibit the activation of inflammation pathways such as nuclear factor kappa B (NF-κB), thus reducing the production of key pro-inflammatory cytokines like tumor necrosis factor-alpha (TNF-α) and interleukin-6 (IL-6), alleviating the inflammatory response (43, 45). Third, butyrate, propionate, and other SCFAs are the primary energy sources for colonic epithelial cells, crucial for maintaining the integrity of the intestinal epithelial barrier and enhancing tight junction functions (46). In this study, the substantial reduction in SCFAs (notably propionate and butyrate) in COVID-19-infected dialysis patients likely impairs these protective mechanisms, increasing intestinal permeability, facilitating lipopolysaccharide (LPS) translocation, inducing metabolic endotoxemia, and triggering a self-amplifying cytokine cascade that exacerbates systemic inflammation and worsens clinical outcomes (47, 48). In contrast, BCFAs, which are mainly generated from BCAA fermentation by proteolytic anaerobes, are comparatively less well characterized than straight-chain SCFAs, and the evidence for their immunomodulatory benefits appears more context dependent across studies (49). In COVID-19, SARS-CoV-2–associated gut dysbiosis has been consistently reported across cohorts, featuring reduced microbial diversity and broad taxonomic and functional shifts that relate to disease severity (50–52). In uremic states, pre-existing disruption of the gut–kidney axis and microbially derived metabolite remodeling in CKD can further constrain beneficial fermentation outputs and amplify inflammatory tone (53). Collectively, these findings indicate that microbiota-derived SCFA loss in uremic patients removes a critical restraint on mucosal and systemic inflammation, synergistically exacerbating SARS-CoV-2 pathology.

The profound depletion of fecal SCFAs observed during the acute phase in our study may set a pathological trajectory for post-COVID-19 metabolic sequelae. Although we did not longitudinally track fecal SCFA levels, emerging multi-omics evidence suggests that such gut microbiome dysbiosis often persists long after viral clearance (54). Specifically, studies have shown that the loss of SCFA-producing bacteria in COVID-19 patients is concurrently associated with impaired microbial biosynthesis of essential amino acids, particularly L-isoleucine, and these alterations correlate with systemic inflammation and disease severity (55). This provides a plausible mechanistic explanation for our serum metabolomics findings in the Long COVID cohort, where we observed persistent perturbations in valine, leucine and isoleucine biosynthesis pathways despite the resolution of acute infection. In dialysis patients, who already suffer from uremic dysbiosis, the acute loss of SCFAs may remove a critical restraint on gut permeability and inflammation, making the gut ecosystem difficult to recover (56). The resulting inability of the microbiome to synthesize sufficient essential amino acids and provide energy substrates likely contributes to the metabolic malnutrition state. This aligns with the clinical symptoms of fatigue and muscle weakness prevalent in our LC patients. Therefore, even without direct fecal sampling in the follow-up period, the acute SCFA crash we observed signals a need for early gut-targeted interventions to potentially prevent these long-term metabolic deficits.

The metabolic reprogramming induced by COVID-19 is likely exacerbated in dialysis patients because it is superimposed on pre-existing uremic malnutrition and metabolic inflexibility (57). In our cohort, non-targeted serum metabolomics identified 54 key differential metabolites, with pathway enrichment analyses indicating that amino acid metabolism was most prominently and extensively perturbed. Notably, glutamine and aspartate—central precursors for purine and pyrimidine nucleotide synthesis—were markedly reduced, consistent with intense utilization to support both viral replication and proliferating immune cells (58, 59). At the same time, tryptophan and sulfur-containing amino acids such as methionine and cysteine were depleted, changes that are expected to promote immunoregulatory kynurenine pathway activity and to limit glutathione synthesis, thereby weakening antiviral immunity and antioxidant defenses (60–62). In parallel, the accumulation of purine degradation products and organic acids suggests enhanced purine catabolism and a shift toward glycolytic energy metabolism in the setting of systemic inflammation and tissue hypoxia. Together, these COVID-19–driven metabolic changes, acting on the background of chronic protein–energy wasting in dialysis patients, likely create a synergistic disturbance that contributes to more severe inflammatory states and poorer clinical outcomes.

This study compared the serum metabolomics between the LC group and the non-LC group, revealing a more persistent metabolic dysregulation pattern. Interestingly, LC patients also exhibited disruptions in amino acid metabolism pathways, including arginine biosynthesis and tyrosine metabolism, with significant reductions in the levels of glutamine, aspartate, and tyrosine in their serum. These metabolic features were highly similar to those observed in the dialysis infection group. This suggests that the onset of LC is not coincidental but results from an imbalance in the amino acid metabolism homeostasis in the patients, which has not recovered in parallel with the alleviation of clinical symptoms. Importantly, this specific imbalance in amino acid metabolism provides a potential metabolic mechanism to explain typical LC symptoms, such as memory decline and susceptibility to fatigue. Specifically, glutamine, as an important energy substrate in the brain and a precursor of the neurotransmitters glutamate and γ-aminobutyric acid (GABA), may lead to mental fatigue and cognitive decline when deficient, as it affects neuronal energy metabolism (63). N-Methyl-D-aspartate (NMDA) receptors, crucial for synaptic plasticity and learning and memory, rely on aspartate, which is involved in the urea cycle and serves as a precursor for NMDA receptor agonists. The depletion of aspartate may impair memory formation (64). Tyrosine, an essential precursor for the synthesis of dopamine and norepinephrine, plays a central role in maintaining attention, alertness, and working memory (65). A deficiency of tyrosine in LC patients can limit the synthesis of these neurotransmitters, directly leading to fatigue and memory impairment. Recent LC multi-omics studies in the general population (66, 67) have also identified persistent disturbances in amino acid and energy metabolism that associate with fatigue, reduced exercise tolerance and neurocognitive complaints, suggesting that our findings in dialysis patients reflect a broader pathophysiological pattern.

In light of the marked depletion of fecal SCFAs (particularly propionate and butyrate) and perturbations in amino acid metabolism observed in our cohort, targeted modulation of the gut microbiota–SCFAs axis and optimization of amino acid and energy metabolism represent conceptually promising adjunctive strategies for dialysis patients with COVID-19 or long COVID. In the context of kidney diseases reduced SCFA production due to gut dysbiosis is linked to aggravated systemic inflammation, immune dysregulation, oxidative stress, and progression of renal injury; conversely, restoration or supplementation of SCFAs has demonstrated renoprotective potential in preclinical models of CKD, diabetic nephropathy, acute kidney injury, and hypertensive nephropathy (68). Direct clinical evidence in maintenance hemodialysis patients further supports feasibility: a pilot study showed that oral sodium propionate supplementation significantly reduced pro-inflammatory markers (CRP, IL-2, IL-17), oxidative stress, gut-derived uremic toxins (indoxyl sulfate, p-cresyl sulfate), and improved insulin resistance, iron metabolism, and quality of life (69). These effects align with the known actions of SCFAs and may be particularly relevant for mitigating COVID-19-associated cytokine dysregulation, metabolic endotoxemia, and uremic amplification in dialysis patients (70). Nevertheless, important challenges persist. No studies have specifically assessed SCFA supplementation in dialysis patients during or after SARS-CoV-2 infection. Potential concerns include gastrointestinal tolerability, optimal dosing to prevent excessive acidification or metabolic overload in the setting of impaired renal clearance, and altered SCFA handling in uremia. Similarly, targeted amino acid optimization (e.g., glutamine or branched-chain amino acids) could theoretically counteract catabolic stress and support immune/neurotransmitter functions, but carries risks of exacerbating uremic toxicity, acid-base imbalance, or hyperammonemia in this population, with currently only indirect evidence available.

In conclusion, modulation of the gut microbiota–SCFAs axis holds translational promise, underpinned by mechanistic and preclinical insights in kidney diseases and preliminary clinical data in hemodialysis. Future randomized controlled trials are essential to determine its safety, efficacy, optimal formulations, timing, and dosing in dialysis patients recovering from COVID-19, thereby translating these metabolic findings into targeted interventions to attenuate acute severity and long-term sequelae in this high-risk group.

## 5 Limitations

First, although 81 maintenance dialysis patients were enrolled, the effective sample size for omics analyses was modest, and the fecal and serum biospecimen sets were only partially overlapping. This may have reduced statistical power and limited the depth of microbiome–metabolome integration. Second, while the enrichment of essential amino acid pathways strongly suggests a microbial contribution to the serum metabolome, the mechanisms by which COVID-19 perturbs the metabolic milieu of dialysis patients are likely to be highly complex. The direct causal flux from gut microbiota to circulating metabolites remains to be fully clarified. Future studies should therefore integrate multi-omics and experimental approaches, expand to multicenter cohorts with larger sample sizes, and incorporate longitudinal sampling at multiple time points. Such efforts will be essential to delineate how COVID-19 influences metabolism in dialysis patients, to define the causal roles of key metabolites in disease progression, and to rigorously assess their translational potential.

## 6 Conclusion

This study demonstrates that COVID-19 infection significantly exacerbates metabolic disturbances in dialysis patients mediated by a disrupted gut-kidney axis, particularly highlighted by the comprehensive depletion of SCFAs and widespread disruption of serum amino acid metabolic pathways. The reduction in SCFAs may weaken their anti-inflammatory and barrier protective functions, thus intensifying systemic inflammation. Meanwhile, the reprogramming of amino acid metabolism provides the necessary material foundation for viral replication while further impairing the host's immune response. Together, these factors interact with the pre-existing metabolic imbalance in dialysis patients, creating a “synergistic effect” that ultimately leads to more severe inflammatory states and adverse clinical outcomes. This study reveals the disrupted characteristics of dialysis patients after COVID-19 infection at the metabolite level, offering a new metabolic perspective on understanding their pathogenesis. Some patients continue to experience persistent amino acid metabolism imbalance after recovery, forming the metabolic basis for LC symptoms. These findings comprehensively uncover the pathological continuity from acute infection to LC in dialysis patients from a metabolic perspective, providing new insights into understanding the clinical heterogeneity of this population and developing targeted intervention strategies focused on restoring gut homeostasis and metabolic balance.

## Data Availability

The original contributions presented in the study are included in the article/supplementary material, further inquiries can be directed to the corresponding author/s.
